# A novel early onset phenotype in a zebrafish model of merosin deficient congenital muscular dystrophy

**DOI:** 10.1371/journal.pone.0172648

**Published:** 2017-02-27

**Authors:** Sarah J. Smith, Jeffrey C. Wang, Vandana A. Gupta, James J. Dowling

**Affiliations:** 1 Departments of Pediatrics and Molecular Genetics, University of Toronto, Toronto, Canada; 2 Division of Genetics, Department of Medicine, Brigham and Women’s Hospital, Harvard Medical School, Boston, MA United States of America; 3 Division of Neurology and Program for Genetics and Genome Biology, Hospital for Sick Children, Toronto, Canada; University of Minnesota Medical Center, UNITED STATES

## Abstract

Merosin deficient congenital muscular dystrophy (MDC1A) is a severe neuromuscular disorder with onset in infancy that is associated with severe morbidities (particularly wheelchair dependence) and early mortality. It is caused by recessive mutations in the *LAMA2* gene that encodes a subunit of the extracellular matrix protein laminin 211. At present, there are no treatments for this disabling disease. The zebrafish has emerged as a powerful model system for the identification of novel therapies. However, drug discovery in the zebrafish is largely dependent on the identification of phenotypes suitable for chemical screening. Our goal in this study was to elucidate novel, early onset abnormalities in the candyfloss (*caf*) zebrafish, a model of MDC1A. We uncovered and characterize abnormalities in spontaneous coiling, the earliest motor movement in the zebrafish, as a fully penetrant change specific to *caf* mutants that is ideal for future drug testing.

## Introduction

Merosin deficient congenital muscular dystrophy (or MDC1A) is a severe muscle disease that is estimated to affect between one to nine-thousand births worldwide, and is the most prevalent CMD in Western countries[1,2]. It is an autosomal recessive disorder caused by mutations in *LAMA2*, the gene that codes for the laminin-α2 protein (formerly known as merosin)[3–6]. Laminins are heterotrimeric extracellular adhesion molecules composed of an α-chain, β-chain, and γ-chain and are temporally and spatially expressed in the basement membranes surrounding various cell types. In skeletal muscle, the laminin-211 isoform (composed of laminins α-2, β-1 and γ-1) predominates[7] and is one of the critical proteins that participates in anchoring the inner actin cytoskeleton to the extracellular matrix (ECM)[8]. Mutations in *LAMA2* result in either a complete absence or severe reduction of laminin-211, which threatens the integrity of the skeletal muscle fiber, ultimately leading to muscle degeneration and cell death.

MDC1A is a clinically devastating disease characterized by severe hypotonia, muscle weakness, elevated creatine phosphokinase levels, joint contractures, white matter abnormalities, and delayed motor milestones, with symptoms presenting at or shortly after birth [9,10]. Less than one quarter of patients achieve independent ambulation [11], and complications include scoliosis, feeding difficulties, wheelchair dependence, and premature mortality: 30 percent of patients die within their first decade of life, most commonly due to respiratory tract infection [2,9]. As there are no curative or significant disease modifying therapies for MDC1A, there is a critical need to identify potential treatments to improve the quality of life of patients with the ultimate goal of curing the disease.

*Danio rerio* (zebrafish) has emerged as an excellent model for the study of human muscle disease [12]. Due to the high fecundity of zebrafish and the rapid *ex utero* development and optical transparency of developing embryos, the zebrafish lends itself to large-scale phenotypic screens of chemical libraries and is thus an ideal model organism for therapy development [13,14]. One of the keys to successful drug discovery in a model organism is identifying suitable phenotypes for compound testing. In the *candyfloss* (*caf*) [15] model, muscle fibers begin detaching and degenerating at 36 hpf, but visualizing this phenotype requires confocal microscopy until approximately 2 dpf when muscle damage can be viewed under plane polarized light as a reduction or absence of birefringence [16]. In this study, we sought to determine whether we could identify a new phenotype that would allow for earlier detection of *caf* mutants, prior to the onset of muscle fiber detachment. Because MDC1A is a muscle disease, we looked at the earliest skeletal movement performed by developing embryos: spontaneous coiling. Zebrafish embryos begin to coil spontaneously at 17 hpf and by 21 hpf, they begin to coil in response to touch [17]. We found that as early as 23 hpf, *caf* mutants have a defect in their coiling abilities after manual dechorionation, which we believe could be exploited as an early phenotypic marker of mutants in large-scale drug screens. Moreover, a coiling defect was not observed in the zebrafish model of Duchenne muscular dystrophy, *sapje* [18], suggesting that this phenotype is not common to all zebrafish models of muscular dystrophies.

## Materials and methods

### Zebrafish husbandry

Heterozygous (*lama2*^*+/-*^*) candyfloss (caf)* and *sapje (sap)* zebrafish were obtained from the University of Tübingen. Both strains are housed and bred in adherence to zebrafish husbandry protocols approved by the Animal Care Committee at the Peter Gilgan Centre for Research and Learning at the Hospital for Sick Children, including specific IACUC approval for the experimentation described in this study (protocol number 29161). All procedures for *lama2*^*cl501*^ fish line were approved from the Harvard University Institutional Animal Care and Use Committee (2016N000304).

### Coiling assay

Embryos from heterozygous matings were collected at 1 hpf and incubated at 28.5°C in system water with methylene blue. At 22 hpf, embryos from all clutches were pooled. At the designated time point (22 23, or 24 hpf), embryos were removed from incubation and their coils were counted either for 15 seconds immediately after dechorionation with forceps or for 30 seconds while remaining in their chorions. Embryos were viewed using an Olympus SZX7 microscope. A full coil was determined to be where the embryo’s tail was able to completely curve around its trunk and touch its head. A partial coil was determined to be when an embryo made the movement to complete a coil, but was unable to. After the coils of an embryo counted, it was placed into an individual well of a 24-well plate filled with system water with methylene blue (egg water) and was incubated at 28.5°C until 3 dpf (*cafs*) or 5 dpf (*sapje*) upon which the genotypes were confirmed with birefringence.

### Birefringence

Birefringence was observed by light microscopy using a plane-polarizing filter on an Olympus SZX7 microscope.

### Statistical analysis

All statistical analyses (student T-test and two-way ANOVA) were completed with Prism 6.0.

## Results

### Defining spontaneous coiling parameters in *caf* zebrafish

**Fig 1:**
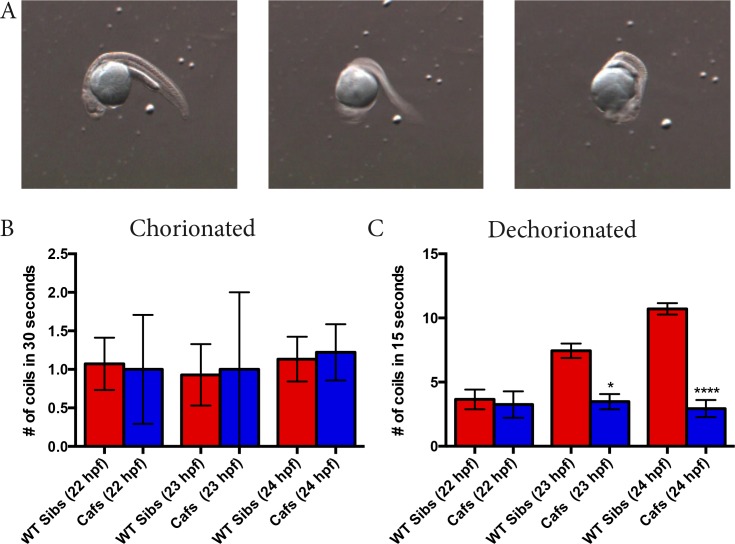
*Caf* mutants display a coiling phenotype as early as 23 hpf. **A**) One full coil completed by a 24 hpf wild type (*lama2*^*+/+*^ and *lama2*^*+/-*^; WT) zebrafish embryo. **B)** Number of coils of WT siblings and *caf (lama2*^*-/-*^*)* mutants over 30 seconds when left in their chorions. There is no significant difference in the coiling abilities of *caf* mutants and their WT siblings at 22 hpf (WT sibs: 1.071 ± 0.3391, n = 14; *cafs*: 1.000 ± 0.7071, n = 4; p>0.9999), 23 hpf (WT sibs: 0.9286 ±0.3987, n = 14; *cafs*: 1.000 ± 1.000, n = 2; p>0.9999) or 24 hpf (WT sibs: 1.133 ± 0.2906, n = 15; *cafs*: 1.222 ± 0.3643, n = 9; p>0.9999). **C)** Number of coils of WT siblings and *caf* mutants in the 15 seconds immediately after manual dechorionation. At 22 hpf, there was no significant difference in number of coils completed by WT siblings and *caf* mutants (WT sibs: 3.654 ± 0.7685, n = 26; *cafs*: 3.250 ± 1.031, n = 8; p>0.9999). However, *cafs* completed significantly less coils than their WT sibs at 23 hpf (WT sibs: 7.444 ± 0.5587, n = 72; *cafs*: 3.476 ± 0.5921, n = 21; p = 0.0194) and 24 hpf (WT sibs: 10.71 ± 0.4436, n = 163; *cafs*: 2.936 ± 0.6646, n = 47; p<0.0001). Bars represent mean ± SEM.

**Fig 2:**
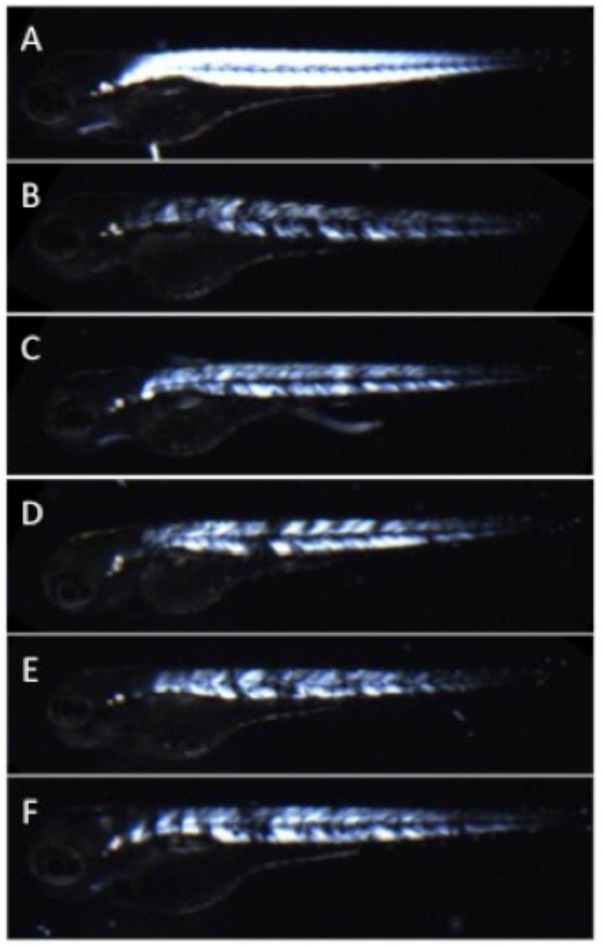
Muscle fiber detachment of *caf* mutants can be observed with birefringence. Under plane polarized light, muscle from WT siblings (A) appears uniformly bright and white, consistent with normal muscle organization. In contrast, *Caf* mutants (B-F_ can be identified as having stochastic patterns of muscle degeneration and detachment with birefringence as early as 2 dpf. This is seen in the muscle compartment as dim white areas (thinned or atrophied fibers) and black spots (presumed areas of muscle fiber detachment). Of note, genotype for all depicted animals was confirmed by Sanger sequencing. Bars represent mean ± SEM.

This study began when we noticed that qualitatively, we were able to predict *caf* mutants based on how often they coiled after manual dechorionation at 24 hours post fertilization (hpf). This led us to design a protocol that would allow for quantitatively measuring the number of coils performed by *caf* (*lama2*^*-/-*^*)*embryos and their wild type (WT) siblings in a short time period (15 or 30 seconds). Heterozygous (*lama2*^*+/-*^*)* zebrafish were mated and embryos were pooled at 22 hpf. At this time point, we were unable to visualize (using birefringence analysis) any muscle fiber detachment and could not visually distinguish *caf* mutants apart from their WT (*lama2*^*+/-*^ and *lama2*^*+/+*^*)* siblings. At 22, 23, and 24 hpf, we counted the number of full coils completed by each embryo within the 15 seconds immediately after manual dechorionation with forceps, or in 30 second period with no dechorionation (i.e. embryos remaining in their chorions). We doubled the time for counting coils when the embryos remained in their chorions because we predicted (based on previous work) that the embryos complete fewer coils if not mechanically stimulated [17]. We determined a full coil to be where the end of an embryo’s trunk was able to curl around and touch its head (Fig 1A). After the coils of an embryo were counted, the embryo was placed into an individual well of a 24-well plate and the embryos developed until a time point where their genotype (*caf* or WT sibling) could be determined with birefringence (Fig 2). Of note, there is complete correspondence between abnormal birefringence and the *caf* genotype. Additionally, in a subset of embryos (n = 40), genotypes were validated by genetic analysis.

### *Caf* mutants complete significantly less coils than WT siblings after manual dechorionation

In embryos derived from a heterozygous (*lama2*^*+/-*^ x *lama2*^*+/-*^*)* mating pair confined in their chorions, we found that there was no significant difference in the coiling abilities of WT siblings and *caf* mutants at any time point (22, 23, and 24 hpf); all embryos would complete approximately one coil within a 30-second time period (Fig 1B). While it is possible that *cafs* and WTs would differ in coiling in their chorions if examined for longer time periods, we did not further investigate this possibility because we were interested in identifying phenotypes suitable for large-scale drug screening.

**Fig 3:**
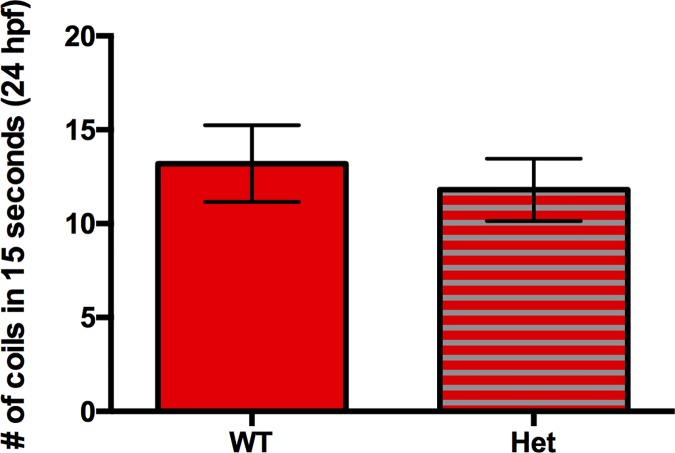
Heterozygous *lama2*^*+/-*^ embryos have no coiling deficiency. Heterozygous (*lama2*^*+/-*^*)* embryos do not display any coiling deficiency compared to their *lama2*^*+/+*^ WT siblings at 24 hpf after manual dechorionation (WT: 13.20 ± 2.037, n = 10; Het: 11.80 ± 1.654, n = 15; p = 0.5984). Bars represent mean ± SEM.

It has been shown that zebrafish embryos vigorously coil in response to touch starting at 21 hpf [17,19]; therefore, we reasoned that stimulating the embryos with manual dechorionation would increase the number of coils they complete, making it more feasible to identify mutants. At 22 hpf, we found that there was no significant difference in the coiling abilities of WT sibs and *caf* mutants upon manual dechorionation. However, we observed that *caf* mutants complete significantly fewer coils than their WT siblings at 23 and 24 hpf (Fig 1C) (S1 and S2 Videos). Furthermore, there is no significant difference in the coiling abilities of *lama2*^*+/-*^ WT embryos (i.e. heterozygotes) compared to *lama2*^*+/+*^ WTs, and *caf* mutants complete significantly less coils than both groups (Fig 3). This novel coiling discrepancy of *caf* mutants presents us with an easily measurable phenotype that has previously never been reported, and thus a protocol that can be easily optimized to act as a read-out in high-throughput drug screens.

### The *lama2*^*cl501*^ line also displays a coiling defect

To validate our observation, we examined another zebrafish line that models merosin deficient congenital muscular dystrophy. This line, *lama2*^*cl501*^, was identified from an ENU screen [20]. It carries a splice site mutation in *lama2* and produces a phenotype indistinguishable from *caf*. Using similar methodology that described above, we measured coiling, both in the chorion and upon dechorionating, at 22, 23, and 24 hpf. As with the *caf* mutants, *lama2*^*cl501*^ mutants also displayed a significant defect in spontaneous coiling that was present at 23 hpf and 24 hpf (S1 Table and S1 Fig). This independent observation supports our conclusion that the coiling defect we observe is due to the *lama2* mutation.

### The coiling defect of *caf* mutants is due likely due to muscular abnormalities

**Fig 4:**
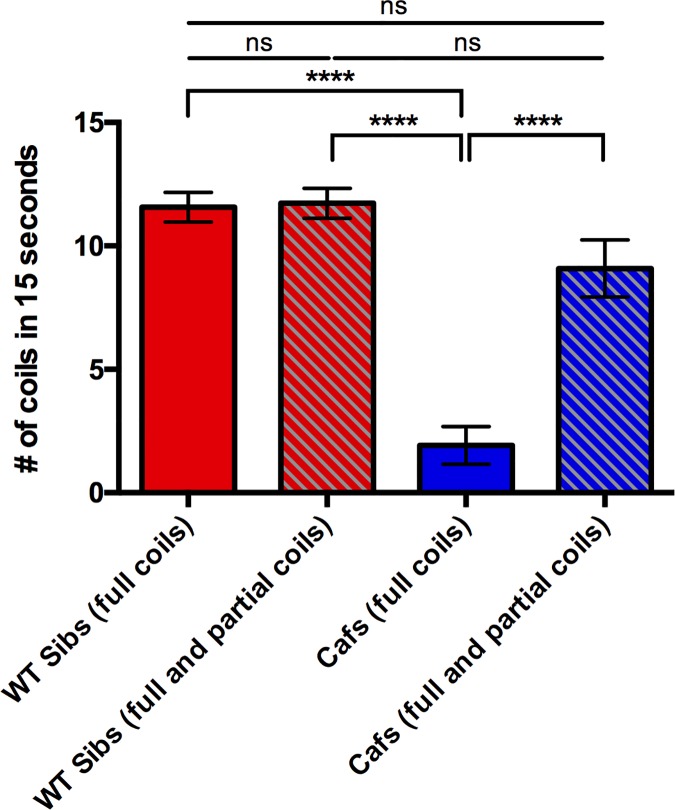
*Caf* mutants attempt to coil as often as their WT siblings. There is no significant difference between the number of full coils and the number of full and partial coils completed by WT sibs, indicating that nearly all of the coiling attempts completed by WT sibs result in a full coil (WT Sibs (full coils): 11.86 ± 0.7253, n = 44; WT Sibs (full and partial coils): 12.02 ±0.6962, n = 44; p = 0.9975). As expected, *cafs* perform significantly less full coils (1.917 ± 0.7633, n = 12) compared to the number of full coils completed by WT sibs (p<0.0001) and the number of full and partial coils completed by WT sibs (p<0.0001). However, when the number of partial coiling attempts by *caf* mutants is combined with their full coils (9.083 ± 1.158, n = 12), their coiling attempts are restored to WT levels when compared to WT full coils (p = 0.2084) and WT full and partial coils (p = 0.1633). Bars represent mean ± SEM.

The coiling of zebrafish embryos in response to touch requires input from both nervous and muscular systems in order to bring about functional motor behavior [19]. As it has been well established that there are white matter abnormalities in MDC1A patients, the coiling discrepancy could be due to defects in both neurological and/or muscular systems. We reasoned that if the coiling defect observed in *caf* mutants was due to ultrastructural abnormalities in skeletal muscle alone, the *cafs* would at least initiate a coil as many times as their WT sibling counterparts. To investigate this, we counted the number of full and partial coils made by *caf* mutants compared that to the number of full and partial coils completed by WT siblings. We counted any attempt to coil that did not result in a complete coil as a partial coil. We found that there was no significant difference in the number of total coils (full and partial) attempted by *caf* mutants compared to WT siblings, indicating that impaired neuronal input is unlikely to be the cause of the abnormal coiling phenotype (Fig 4). This is perhaps unsurprising, as innervation in *caf* zebrafish was previously reported to be normal [15]. As such, we believe that the coiling phenotype of *caf* mutants is due to primary defects in muscle fibers that have yet to be elucidated.

### Abnormal filamentous actin distribution supports an early muscle defect in *caf* embryos

**Fig 5:**
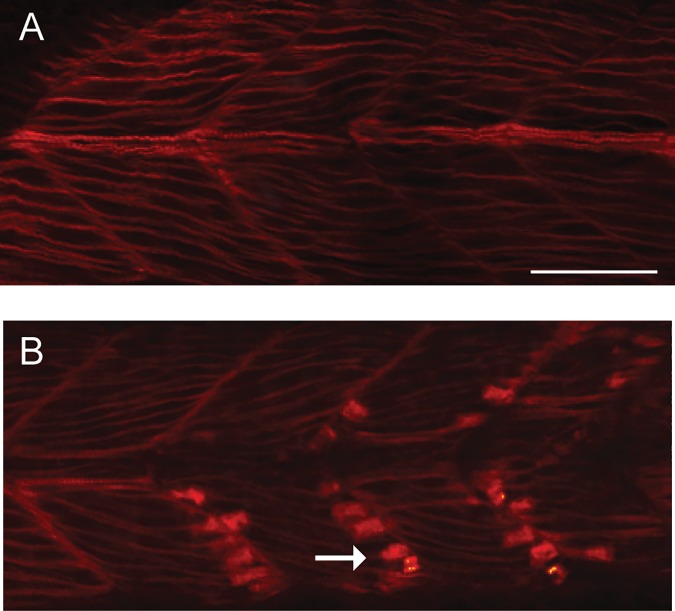
Abnormal phalloidin staining in skeletal muscle from *caf* mutant embryos. Wild type (WT) and *caf* mutants were staining with phalloidin to illuminate filamentous actin and then visualized whole mount by confocal microscopy. (A) WTs show the expected pattern of staining at 24 hpf (n = 5). (B) In muscle from *caf* mutants, there is an accumulation of intense staining in the region of the myotendinous junction (arrow) (n = 5). Scale bar = 10 um.

Our observation of disrupted full coils in 24 hpf dechorionated *caf* embryos suggests that mutant skeletal muscle may be abnormal at this early stage of muscle development. To investigate this further, we utilized phalloidin staining as a means of detecting changes in myofiber organization. Phalloidin highlights filamentous actin and has been used by others as a means of documenting abnormalities in dystrophic muscle of various zebrafish models. Wild type embryos at 24 hpf showed the expected pattern of phalloidin staining (n = 5). *Caf* embryos, in contrast, displayed altered expression, with intense staining detected at a location consistent with the myotendinous junction (n = 5) (Fig 5). While the full significance of this observation merits further investigation, these data further support an early skeletal muscle phenotype in *caf* mutants.

### Coiling deficiency is not common to all zebrafish models of muscular dystrophy

**Fig 6:**
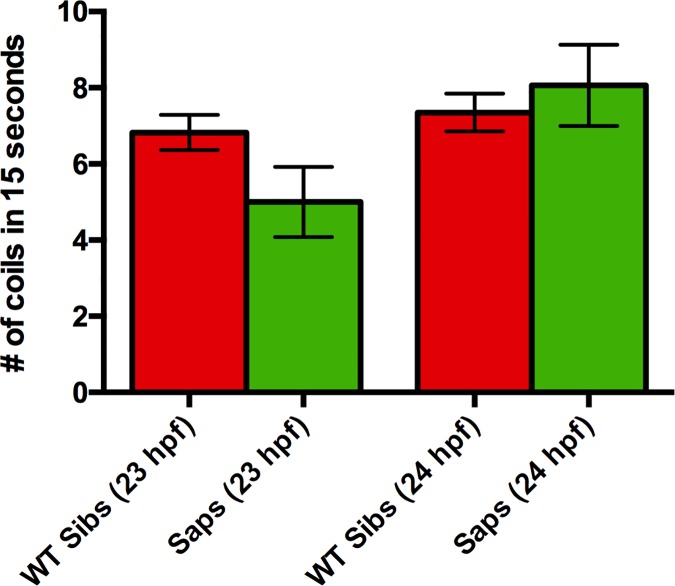
Coiling deficiency is not observed in the zebrafish model of Duchenne muscular dystrophy. *Sapje* mutants, the zebrafish model of Duchenne muscular dystrophy, do not present with a coiling phenotype at 23 hpf (WT sibs: 6.823 ± 0.4642, n = 62; *cafs*: 5.000 ± 0.9244, n = 11; p = 0.1088) or 24 hpf (WT sibs: 7.347 ± 0.4950, n = 72; *cafs*: 8.063 ± 1.066, n = 16; p = 0.9997). Bars represent mean ± SEM.

Because of the significant difference in the coiling abilities of *caf* mutants compared to their WT siblings, we went on to determine whether this phenotype is unique to *caf* mutants by examining whether the zebrafish model of Duchenne muscular dystrophy, *sapje*, had similar defects. Because the spontaneous coiling of *caf* mutants in their chorions was not predictive of their genotype, we only examined the number of coils completed by *sapje* mutants after manual dechorionation. At both 23 and 24 hpf, we found that *sapje* mutants had a similar number of full coils as compared to wild type (Fig 6), indicating that not all zebrafish models of muscular dystrophies a coiling phenotype and suggesting that this phenotype may be unique to *caf* mutants.

## Discussion

In this study, we have further characterized the zebrafish model of MDC1A, *candyfloss* (*caf*), by identifying a novel coiling phenotype. Prior to this study, the first reported pathology of *caf* mutants was slow muscle fiber degeneration at 36 hpf [15]. Here, we have observed a defect in the ability of *caf* mutants to complete full coils, which is mediated primarily by slow muscle fibers [19], as early as 23 hpf, long before the onset of muscle detachment and degeneration. This observation suggests that although the fibers differentiate appropriately and appear normal prior to 36 hpf [15], there may be abnormalities in the muscle that are impairing the ability of the *caf* mutants to properly coil, an assertion supported by our demonstration of abnormal distribution of filamentous actin in 24 hpf myofibers. Furthermore, we did not observe this phenotype in a zebrafish model of Duchenne muscular dystrophy, *sapje*, indicating that this phenotype is not common to all zebrafish models of muscular dystrophy and may be unique to *caf* mutants.

In MDC1A patients, disease symptoms present either at or shortly after birth, and it is speculated that these patients are never truly pre-symptomatic. In contrast, the onset of Duchenne muscular dystrophy is later, where boys begin developing symptoms between 1.5–2.5 years of age. The reason as to why MDC1A is a congenital muscle disease is incompletely understood. Mehuron et al identified early perinatal pathology in a mouse model of MDC1A, and correlated this with increases in apoptosis during myogenesis [21]. Our findings of a coiling defect in *caf* mutants are consistent with the congenital symptoms seen in both patients and mice with MDC1A, and they open an avenue for further exploration of the mechanisms underlying the early-onset phenotype.

Importantly, the phenotype we have identified, because of the ease in its identification and the ability to automate the analysis using recording systems such as the Viewpoint Zebrabox, can be exploited for large-scale drug screens. This phenotype holds advantages over birefringence, the tool most commonly used for drug development in dystrophic models. One advantage is that the early coiling phenotype occurs prior to muscle detachment, and thus may represent a treatment window period. It is reasonable to speculate that, at least in zebrafish, fully detached muscle will be difficult to “fix” with chemical modification, and thus limit the opportunity for finding drug targets using this model. Additionally, using the coiling phenotype as a measure of therapeutic effectiveness rather than the dystrophic phenotype, which is fully penetrant at 3 dpf [15], is a more cost-effective approach, as less chemicals would be required over time.

In conclusion, we have identified a novel coiling phenotype of *caf* mutants suitable for use in high-throughput drug screens that may serve as a measure of therapeutic effectiveness by harnessing the power of automated movement-tracking systems such as the Zebrabox platform (Viewpoint) or Noldus. This strengthens the characterization of the *caf* zebrafish model of MDC1A and lays the foundation for further experiments that aim to understand the mechanisms that result in stochastic muscle fiber degeneration in MDC1A and other congenital muscular dystrophies.

## Supporting information

S1 Table Coiling in the *lama^2cl501^* zebrafish model of MDC1A. 1. Number of coils in the chorion, wild type versus *lama2*^*cl501*^mutants. There was no statistical difference in coiling behavior between the two groups when embryos remained in their chorions. 2. Number of coils for dechorionated embryos, wild type versus *lama2*^*cl501*^ mutants. There was a statistically significant decrease in coiling by *lama2*^*cl501*^ mutants in embryos dechorionated at 24 hours post fertilization but not at time points before this.
(DOCX)

S1 Fig *lama^2cl501^* mutants have reduced coiling upon dechorionation. Still images from time lapse videos of wild type clutchmates (control) and *lama2*^*cl501*^ (*lama2*) mutants at 24 hours post fertilization. Videos were taken just after dechorionation. *Lama2* mutants demonstrate only partial coiling and do not complete a normal/full coiling in the 2 second period shown. In contrast, the control embryo completes 2 full coils.
(DOCX)

S1 Video Spontaneous coiling of a dechorionated wild type embryo at 24 hours post fertilization. (MOV)

S2 Video Spontaneous coiling of a dechorionated *caf* embryo at 24 hours post fertilization. (MOV)
