# Supplementary material for: A novel early onset phenotype in a zebrafish model of merosin deficient congenital muscular dystrophy
Source: PLoS One. 2017 Feb 27;12(2):e0172648. doi: 10.1371/journal.pone.0172648 (PMC5328290; doi:10.1371/journal.pone.0172648)
Supplement: S1 Table — 1. Number of coils in the chorion, wild type versus lama2cl501mutants. There was no statistical difference in coiling behavior between the two groups when embryos remained in their chorions. 2. Number of coils for dechorionated embryos, wild type versus lama2cl501 mutants. There was a statistically significant decrease in coiling by lama2cl501 mutants in embryos dechorionated at 24 hours post fertilization but not at time points before this. (DOCX) [file pone.0172648.s001.docx]

# *Supplemental Table I: Coiling in the lama2^cl501^ zebrafish model of MDC1A*

# Number of coils in the chorion

| Time Point (h) | Genotype | Coils/30 seconds | n |
| --- | --- | --- | --- |
| 22 | Wild-type | 1.251± 0.480 | 12 |
|  | *lama2* | 1.192± 0.388 | 12 |
| 23 | Wild-type | 1.103± 0.323 | 12 |
|  | *lama2* | 1.162± 0.457 | 12 |
| 24 | Wild-type | 1.051± 0.411 | 12 |
|  | *lama2* | 1.117± 0.307 | 12 |

# Number of coils for dechorionated embryos

| Time Point (h) | Genotype | Coils/15 seconds | n |
| --- | --- | --- | --- |
| 22 | Wild-type | 2.92 ± 0.712 | 12 |
|  | *lama2* | 2.79 ± 0.689 | 12 |
| 23 | Wild-type | 5.93 ± 1.838 | 12 |
|  | *lama2* | 4.27± 0.972 | 12 |
| 24 | Wild-type | 8.39± 0.943 | 12 |
|  | *lama2* | 3.92± 0.429 | 12** |

# **p<0.05 (as compared to wild type)
