# Supplementary material for: A novel early onset phenotype in a zebrafish model of merosin deficient congenital muscular dystrophy
Source: PLoS One. 2017 Feb 27;12(2):e0172648. doi: 10.1371/journal.pone.0172648 (PMC5328290; doi:10.1371/journal.pone.0172648)
Supplement: S1 Fig — Still images from time lapse videos of wild type clutchmates (control) and lama2cl501 (lama2) mutants at 24 hours post fertilization. Videos were taken just after dechorionation. Lama2 mutants demonstrate only partial coiling and do not complete a normal/full coiling in the 2 second period shown. In contrast, the control embryo completes 2 full coils. (DOCX) [file pone.0172648.s002.docx]

**Supplemental Figure 1: *lama2^cl501^* mutants have reduced coiling upon dechorionation**

Still images from time lapse videos of wild type clutchmates (control) and *lama2^cl501^* (*lama2*) mutants at 24 hours post fertilization. Videos were taken just after dechorionation. *Lama2* mutants demonstrate only partial coiling and do not complete a normal/full coiling in the 2 second period shown. In contrast, the control embryo completes 2 full coils.
